# Editorial: Mitochondrial dysfunction in inflammation and autoimmunity

**DOI:** 10.3389/fimmu.2023.1304315

**Published:** 2023-10-04

**Authors:** Jens Staal, Luz Pamela Blanco, Andras Perl

**Affiliations:** ^1^ Unit of Molecular Signal Transduction in Inflammation, Flemmish Institute of Biotechnology (VIB)-UGent Center for Inflammation Research, Ghent, Belgium; ^2^ Department of Biomedical Molecular Biology, Ghent University, Ghent, Belgium; ^3^ Department of Biochemistry and Microbiology, Ghent University, Ghent, Belgium; ^4^ The National Institute of Arthritis and Musculoskeletal and Skin Diseases (NIAMS), National Institutes of Health (NIH), Bethesda, MD, United States; ^5^ Department of Medicine, Upstate Medical University, Syracuse, NY, United States; ^6^ Department of Biochemistry and Molecular Biology, Upstate Medical University, Syracuse, NY, United States; ^7^ Department of Microbiology and Immunology, Upstate Medical University, Syracuse, NY, United States

**Keywords:** inflammation, mitochondria, autoimmune diseases, ferroptosis, interferonopathy, metabolism, ROS - reactive oxygen species, DAMP (damage-associated molecular pattern)

Mitochondria are the remnants of the ancient endosymbiont bacteria that together with a cell related to the asgard archaea formed the original ur-eukaryote (1). Mitochondria are however best known as the powerhouses responsible for energy production in eukaryotic cells, and they play a pivotal role in maintaining cellular and organismal health, and metabolic homeostasis. Any compromise in their functionality can have far-reaching consequences.

Notably, dysfunctional mitochondria are frequently associated with the excessive generation of intracellular reactive oxygen species (ROS), leading to a state of enhanced oxidative stress. This oxidative stress is not only detrimental to the mitochondria themselves, but also initiates destructive cascades, impacting the entirety of the cellular machinery and creating pathological feedback loops, such as the immunogenic cell death via ferroptosis (2); further exacerbating the tissue damage inflicted. Indeed, mitochondrial ROS-induced damage, cell death, and subsequent immunogenicity are significant drivers onto several autoimmune diseases and could thus potentially be a therapeutic target, for example in rheumatoid arthritis as discussed by Jing et al. By contrast, increased resistance to oxidative stress due to overexpression of glucose 6-phosphate dehydrogenase has been implicated in the causation and flare of rheumatoid arthritis (3). Alternatively, mitochondrial oxidative stress is a driver of lupus (4), which is responsive to treatment with antioxidants, such as acetylcysteine (5).

Dysfunctional mitochondria can release mitochondrial-derived nucleic acids into both the intracellular and extracellular milieu. This phenomenon holds the potential to amplify pro-inflammatory type I interferon (IFN) responses, a prominent feature in certain autoimmune disorders (6) — so-called interferonopathies. Furthermore, these malfunctioning mitochondria may serve as a source of modified self-antigens, commonly referred to as autoantigens, which can contribute to the development of autoimmune conditions. In addition, they can release danger-associated molecular patterns (DAMPs), further intensifying the inflammatory response. Examples of these mitochondria-derived DAMPs (mtDAMPs) have been discussed in this Research Topic as a hypothetical driver of Kawasaki disease by Beckley et al. and how mtDAMPs can induce systemic inflammation after local tissue damage were reviewed in this Research Topic by Ye et al.

Notably, malfunctioning mitochondria within crucial immune cells may disrupt the establishment of regulatory networks that are essential for preventing, attenuating, or controlling autoimmune conditions and inflammatory processes. Moreover, metabolic disturbances in non-immune cells can indirectly result in an immune cell phenotype. For example, as demonstrated by a transcriptomic analysis on polycystic ovary syndrome (PCOS) by Chen et al., disturbances in primary metabolism in hormone-producing cells can indirectly result in immune cell activation. Therefore, understanding the intricate interplay between mitochondrial dysfunction and the initiation and perpetuation of inflammation and autoimmunity is of paramount importance.

## Author contributions

JS: Writing – original draft, Writing – review & editing. LB: Writing – review & editing. AP: Writing – review & editing.
